# Multiple Local and Recent Founder Effects of *TGM1* in Spanish Families

**DOI:** 10.1371/journal.pone.0033580

**Published:** 2012-04-12

**Authors:** Laura Fachal, Laura Rodríguez-Pazos, Manuel Ginarte, Jaime Toribio, Antonio Salas, Ana Vega

**Affiliations:** 1 Fundación Pública Galega de Medicina Xenómica-SERGAS, Grupo de Medicina Xenómica-USC, CIBERER, IDIS, Santiago de Compostela, Spain; 2 Department of Dermatology, Complejo Hospitalario Universitario, SERGAS, Faculty of Medicine, Santiago de Compostela, Spain; 3 Unidade de Xenética, Instituto de Ciencias Forenses, Facultade de Medicina, and Departamento de Anatomía Patolóxica e Ciencias Forenses, Facultade de Medicina, Universidade de Santiago de Compostela, Galicia, Spain; Instituto de Higiene e Medicina Tropical, Portugal

## Abstract

**Background:**

Mutations in the *TGM1* gene encoding transglutaminase 1 are a major cause of autosomal recessive congenital ichthyosis. In the Galician (NW Spain) population, three mutations, c.2278C>T, c.1223_1227delACAC and c.984+1G>A, were observed at high frequency, representing ∼46%, ∼21% and ∼13% of all *TGM1* gene mutations, respectively. Moreover, these mutations were reported only once outside of Galicia, pointing to the existence of historical episodes of local severe genetic drift in this region.

**Methodology/Principal Findings:**

In order to determine whether these mutations were inherited from a common ancestor in the Galician population, and to estimate the number of generations since their initial appearance, we carried out a haplotype-based analysis by way of genotyping 21 SNPs within and flanking the *TGM1* gene and 10 flanking polymorphic microsatellite markers spanning a region of 12 Mb. Two linkage disequilibrium based methods were used to estimate the time to the most recent common ancestor (TMRCA), while a Bayesian-based procedure was used to estimate the age of the two mutations. Haplotype reconstruction from unphased genotypes of all members of the affected pedigrees indicated that all carriers for each of the two mutations harbored the same haplotypes, indicating common ancestry.

**Conclusions/Significance:**

In good agreement with the documentation record and the census, both mutations arose between 2,800–2,900 years ago (y.a.), but their TMRCA was in the range 600–1,290 y.a., pointing to the existence of historical bottlenecks in the region followed by population growth. This demographic scenario finds further support on a Bayesian Coalescent Analysis based on *TGM1* haplotypes that allowed estimating the occurrence of a dramatic reduction of effective population size around 900–4,500 y.a. (95% highest posterior density) followed by exponential growth.

## Introduction

Autosomal recessive congenital ichthyosis (ARCI) is a rare, nonsyndromic, heterogeneous disorder of cornification, defined into three clinical subtypes which includes the spectrum of lamellar ichthyosis (LI; OMIM 242300) and congenital ichthyosiform erythroderma (CIE; OMIM 242100) as well as harlequin ichthyosis (HI; OMIM 242500), which has been recently included in this group of disorders [1]. In the majority of patients, ARCI is caused by a transglutaminase 1 deficiency resulting from mutations in both copies of the transglutaminase 1 gene (*TGM1*) on chromosome 14 [2].

During the *TGM1* mutation screening of a Galician (NW Spain) ARCI cohort [3] we identified some frequent mutations in several families and, surprisingly, three of them, namely c.2278C>T (three unrelated families), c.1223_1227delACACA (one family) and c.984+1G>A (one family) were found in a homozygous state. Recently, an additional homozygous family for the c.2278C>T mutation was identified (Family 17, Figure S1). The *TGM1* mutations c.2278C>T, c.1223_1227delACACA and c.984+1G>A were therefore, present in seven, four and two apparently non-consanguineous families, accounting for 45.83%, 20.83% and 12.5% of all *TGM1* mutated alleles, respectively (Table 1).

**Table 1:** Summary of patients and mutation data for c.2278C>T and c.1223_1227delACACA carrier.

Nucleotide change	Amino acid change	N° mutated alleles(%)	Id^a^	Sex^b^	Age^c^	Clinical Diagnosis	Gene	Alleles
c.2278C>T	p.Arg760×	11	1.IV.1	M	2	LI	*TGM1*	c.[2278C>T]+[2278C>T]
		(45.83)	2.IV.4	F	25	LI	*TGM1*	c.[2278C>T]+[2278C>T]
			2.III.5	F	56	LI	*TGM1*	c.[2278C>T]+[2278C>T]
			3.IV.4	F	27	LI	*TGM1*	c.[2278C>T]+[2278C>T]
			17.III.3	M	-	Non-affected (father of deceased LI children)	*TGM1*	c.[2278C>T]+[ = ]
			17.III.4	F	-	Non-affected (mother of deceased LI children)	*TGM1*	c.[2278C>T]+[ = ]
c.1223_1227delACACA	p.Asp408ValfsX21	5	4.III.1	M	1	LI	*TGM1*	c.[1223_1227delACACA]+[2278C>T]
		(20.83%)	5.III.3	F	31	LI	*TGM1*	c.[1223_1227delACACA]+[2278C>T]
			6.IV.2	F	7	LI	*TGM1*	c.[1223_1227delACACA]+[2278C>T]
			7.III.1	M	46	LI	*TGM1*	c.[1223_1227delACACA]+[1223_1227delACACA]
c.984+1G>A	p.Met329_Val330ins10p.Val330MetfsX12	3	9.III.1	F	80	LI	*TGM1*	c.[984+1G>A]+[1187G>A]
		(12.5%)	9.III.2	F	84	LI	*TGM1*	c.[984+1G>A]+[1187G>A]
			10.V.1	F	11	LI	*TGM1*	c.[984+1G>A]+[984+1G>A]

a The first Arabic number indicates the family, the Roman number the generation, and the last Arabic number the affected individual.

b M = male, F = female.

c age at the moment of the study.

**Figure 1:**
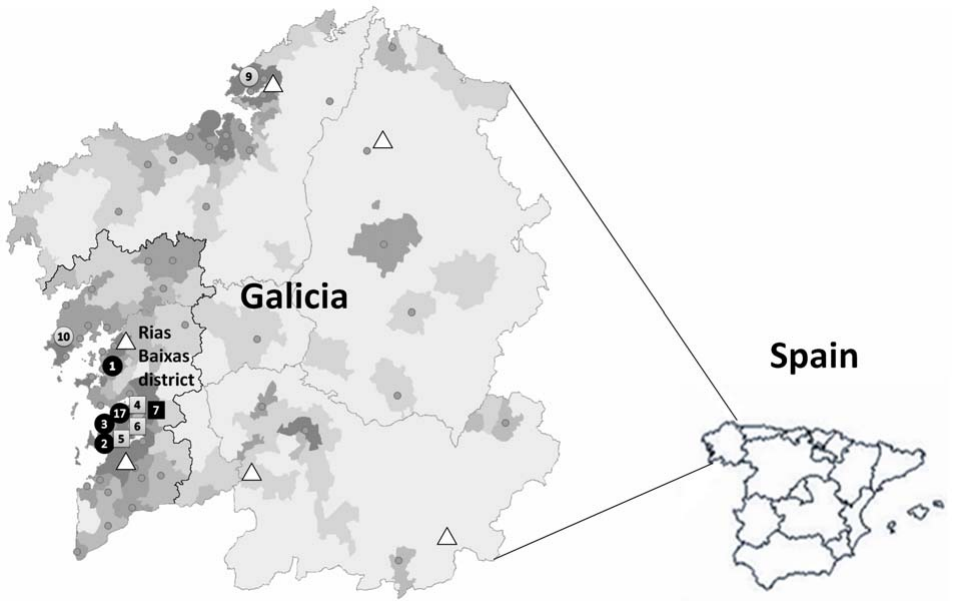
Population density map (in contemporary Galicia) showing the geographic distribution of the Galician ARCI families. Grey dots: built-up areas with 10000 or more inhabitants. Black circles: homozygous c.2278C>T carriers. Black squares: homozygous c.1223_1227delACACA carrier. Grey squares: c.2278C>T/c.1223_1227delACACA carriers. Grey circles: ARCI patients carriers of c.984+1G>A. Triangles: ARCI families harboring other mutations.

The estimated prevalence of ARCI in the USA is 1∶200,000–300,000, while in Europe it has been estimated to be around 1 in 200,000 persons [4]. ARCI seems however to be more frequent in Norway (1∶91,000) owing to founder effects [5]. The estimated prevalence of ARCI in Galicia, with a census population of 2.8 million people, is about 1∶122,000 (23 identified patients) [3]. However, most of the cases were observed in a local coastal Galician district (Figure 1), and therefore, the prevalence of ARCI in this area reaches the highest values reported in the literature to date, namely 1∶33,000 (see below). As in Norway [5], the high prevalence of ARCI in Galicia could have been due to founder effects and/or local consanguinity. Alternatively, the background haplotype structure of the original mutations observed in the Galician population could have favored the presence of mutational hotpots at the *TGM1* gene. It is possible to discriminate between a founder mutation and a mutational hotspot by determining whether a specific mutation arises on a common genetic background or appears to occur independently multiple times. Moreover, in the case of confirming that a mutation is a true founder mutation, the size of the conserved region surrounding the mutation can be used to estimate the age of the mutation. Therefore, the aim of our study was: (i) to determine if the c.2278C>T, c.1223_1227delACACA and c.984+1G>A *TGM1* mutations were inherited from a common ancestor in the Galician population, and (ii) to estimate the number of generations since the appearance of these mutations in our population. The results will be discussed in a demographic context as inferred from the historical record.

## Results

### Mutation c.2278C>T

Seven families were carriers of the c.2278C>T mutation, four of them in a homozygous state (Families 1, 2, 3 and 17; Figures S2, S3, S4 and S1, respectively) while three of them in a heterozygous state (Families 4, 5, 6; Figures S5, S6 and S7, respectively). Two members of Family 2 (Figure S3) suffered from LI, the proband and her maternal uncle. Therefore, three different alleles could be evaluated, two from the proband and one from her maternal uncle (i.e. the second disease allele from the maternal branch not transmitted to the proband by her mother). We found a common 2.6 cM haplotype that included all the *TGM1* intragenic markers and fourteen markers located close to *TGM1* (from D14S1032 to D14S581, Table S1). The control chromosomes displayed a large number of different haplotypes for these markers. However, none of the 200 normal chromosomes shared the patient's haplotype. The remaining six markers diverged into several haplotypes. Two alleles (2.IV.4.A and 17.III.3.A) shared a common haplotype of 15.25 cM (spanning from D14S1060 to D14S742), indicating a possible closer relationship between the different families (Families 2 and 17; Figures S2 and S1, respectively); nevertheless, no familiar relationship was reported in at least three generations.

The markers used for calculating the mutation age are shown in Table 2. The age of the TMRCA of c.2278C>T was estimated to be in the interval of 18 to 51 generations by the Bergman estimator [6] and between 11 and 30 generations by the Risch estimator [7]. Due to the fact that allele 9 of the marker D14S1060 was less common in the disease population (0.25) than among the normal controls (0.16), this marker did not provide any information when the Bergman estimator was used. Similarly, the marker D14S1043 could not be evaluated by the Risch method owing to the fact that the frequency of the allele present in the common region, was the same as the previous neighboring marker. Using the correction proposed by Labuda et al. [8] for the Risch method, the range of the TMRCA increased to 17–44 generations.

**Table 2:** Estimation of the TMRCA and the age (generations) of the c.2278C>T and c.1223_1227delACACA mutations by Risch et al, Bergman et al. and the DMLE methods.

Mutation	Marker	Founder allele	Distance from mutation (Mb)	TMRCA			Time of the mutation
				Bergman estimator	Risch estimator	Risch estimator adjusted by Labuda correction	DMLE
**c.2278C>T**	D14S1043	5	3.34	40	-	-	
	D14S72	3	3.32	65	33	52	
	D14S742	6	2.52	29	29	37	
	D14S275	8	1.98	20	20	31	
	D14S1042	1	4.54	19	13	21	
	D14S1060	9	8.7	-	7	11	
	**Single marker method:** average (95%CI)			35 (18–51)	20 (11–30)	30 (17–44)	
	**DMLE method:** result (95% Bayesian CI)						96 (80–122)
**c.1223_1227delACACA**	D14S1043	3	-	-	-	-	
	D14S72	5	3.33	18	26	45	
	D14S742	5	2.53	24	24	34	
	D14S275	6	1.97	23	23	35	
	D14S1042	3	4.53	20	20	29	
	D14S1060	10	8.69	10	-	-	
	**Single marker method:** average (95%CI)			22 (17–27)	23 (21–25)	36 (28–41)	
	**DMLE method:** result (95% Bayesian CI)						94 (72–124)

TMRCA = Time to the most recent common ancestor.

The age of the c.2278C>T mutation, according to the Bayesian method implemented in DMLE [9], corresponds to approximately 96 generations ago (95% Bayesian CI: 80–122).

### Mutation c.1223_1227delACACA

The *TGM1* mutation c.1223_1227delACACA was found in four families (Families 4, 5, 6, 7; Figures S5, S6, S7, S8, respectively), once in a homozygous state (Family 7, Figure S8). The haplotype in the short conserved genotype (1.33 cM spanning from marker D14S264 to D14S581, Table S1) of five disease chromosomes is rare in a control population (0.02), supporting the idea that it is a common founder mutation.

The markers used for estimating the mutation age are shown in Table 2. The marker D14S1060 could not be used by the Risch estimator owing to the frequency of allele 10, which was the same as the most adjacent marker of the conserved region. The marker D14S1043 was not evaluated by either the Bergman or Risch estimators due to the fact that (i) founder allele 3 was more common in the control (0.73) than in the diseased population (0.4), and (ii) that this allele had the same frequency as the adjacent marker of the conserved region. The TMRCA estimated age of the c.1223_1227delACACA *TGM1* mutation was calculated to be in the range of 17–27 generations by the Bergman estimator and 21–25 generations by the Risch estimator. When the correction proposed by Labuda et al. was used, the generations interval increased to 28–41.

The age of the c.1223_1227delACACA *TGM1* mutation according to the DMLE software was 94 generations (95% Bayesian CI: 72–124).

### Mutation c.984+1G>A

Three patients from two apparently unrelated families were carriers of c.984+1G>A (Families 9 and 10; Figures S9, S10, respectively), one in a homozygous state (Family 10, Figure S10). Haplotype analysis revealed a common haplotype spanning 1.42 cM (from D14S1032 to D14S742, Table S1), absent in the control population. Due to the low number of c.984+1G>A carrying chromosomes, the estimation of the TMRCA and the coalescent age of the mutation could not be investigated.

### Galician demography as inferred from Bayesian Evolutionary Analysis of *TGM1* haplotypes

**Figure 2:**
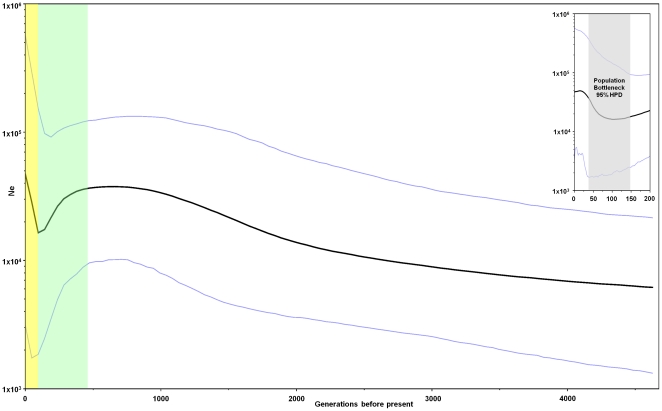
A Bayesian skyline plot derived from a sample of *TGM1* haplotypes of contemporary Galicians. The blue lines represent the 95% HPD (highest posterior density) effective population size (Ne) while the black one provides the mean effective population size through time. Green and yellow backgrounds highlight the main periods of population bottleneck and population expansion regarding the mean effective population size, respectively. The upper-right inset shows the last 200 generations; the grey background highlights the bottleneck period that expand to about 30 generations ago according to the lower 95% HDP.

A Bayesian Coalescence Analysis was carried out using BEAST [10]. The Bayesian skyline plot of Figure 2 shows a demographic model for the Galician population that can be summarized in three main segments: a) a long period of smooth but continuous population growth 150–4,600 generations ago (4,500–13,800 y.a.); b) an historical bottleneck dated 30–150 generations ago (900–4,500 y.a; 95% highest posterior density, HPD); and c) a period of exponential population growth running from about 30 generations ago (900 y.a.) till present.

## Discussion

The present study was stimulated by the fact that Galicia (NW Spain) shows one of the highest prevalence rates of ARCI worldwide, and that the observed mutations in Galician carriers (c.2278C>T, c.1223_1227delACACA and c.984+1G>A) were reported only once outside Galician territory. A better characterization of the mutations responsible for ARCI would also help to design efficient mutational screening procedures and genetic counseling in Galicia.

In order to explore whether the high frequency of the c.2278C>T, c.1223_1227delACACA, and c.984+1G>A *TGM1* mutations in the Galician population were due to a founder effect or any other factor (e.g. mutational hotspot), we searched for evidence of shared common haplotypes in family carriers. Recruited patients were asked about their genealogy. Pedigrees were reconstructed for at least three generations and demographic data were obtained to determine their geographic origin. The demographic distribution within the regional map of Galicia revealed that most of the affected families originate from the same geographical area along the west-central coast, called Rías Baixas. This is today the most densely populated region of Galicia (with a census population size of 750,000 inhabitants). The prevalence of ARCI in this region is therefore the highest observed worldwide in human populations to date.

The results indicate that carriers of the two studied pathogenic mutations descended from a common recent ancestor for each mutation. The TMRCA and the age of the mutation were estimated for two of the mutations. Thus, depending on the method used, the TMRCA of c.2278C>T and c.1223_1227delACACA mutations was calculated to be in the range of 22–43 to 20–33 generations ago (averaging the Bergman's and Labuda's estimates), respectively. The DMLE method identified the time at which the two mutations appeared to be approximately 94–96 generations. Therefore, while the TMRCA dates to about ∼600 to ∼1,290 years ago (y.a.) for the two mutations, the mutational events could be significantly older, namely, 2,800 to 2,900 y.a. Thus, our study points to the existence of strong founder effects occurring in Galicia in recent times. The three studied pathogenic mutations seem to be placed in three different uncommon or inexistent haplotypes in control population, and two of them could be dated as occurring only about 2,800–2,900 y.a. There are two possible scenarios that could explain the results: the *TGM1* mutations c.2278C>T and c.1223_1227delACACA arose in the Galician region about 2,800–2,900 y.a., branching out into unrelated Galician individuals since a population bottleneck before 900 y.a. reduced the haplotype diversity to the two common ancestors of the actual c.2278C>T and c.1223_1227delACACA haplotypes. Alternatively, these two mutations could have been introduced by immigrants arriving to the west-central coast of Galicia about 600–1,290 years ago, branching out into the today's observed haplotypes.

Estimates of the population size of the Galician population 1,000 y.a. are about 232,000 habitants (in contrast to the 2.8 million people today) [11]. Thus, when the mutations first appeared or arrived from other populations, the allele frequency would be about 1/464,000 (0.00022%) for each mutation. The reason for the survival and increase of these mutations to high frequencies in the Galician population could be the congregation of the predominantly rural Galician population into isolated parishes, followed by rapid growth from a limited group of founders. A well-documented abrupt decrease in the number of habitants in the mid-14^th^ century due to famine and the Black Death was probably followed by consecutive cycles of slight decreases and acute increases in population [12]. The most remarkable demographic growth experienced in Galicia occurred during the 16^th^ century, when, according to the census, the population doubled in size [12]. This demographic scenario, coupled with the relative isolation of Galicia from the rest of the Iberian Peninsula (owing to its geographic location and complex orography) have contributed to keep these mutations confined to Galicia for centuries. This marked tendency for isolation coupled with the cultural features of the region (e.g. Galician language) have created a particular genetic identity [13]. The high frequency of Galician founder pathogenic mutations reported to date [14], [15] is also in good agreement with this demographic scenario. Finally, a Bayesian Coalescent analysis carried out on *TGM1* haplotypes yield further support to this demographic scenario. In neighboring regions to Galicia, such as in the Franco-Cantabrian refugee, there are already evidences for such local and also recent (about ∼1,000 y.a.) population expansions as recorded in the mtDNA molecule [16]–[18].

The two Galician mutations c.2278C>T and c.1223_1227delACAC have been reported only once each outside Galicia. Thus, c.2278C>T was identified (together with the known Norwegian mutation c.877-2A>G) in an ‘African-American’ male [19]; whereas the geographical origin of the non-Galician carrier of c.1223_1227delACACA was not specified by the authors [20]. It is important to note that in this last report, patients from diverse ethnic groups, including Portugueses and ‘Hispanics’ were analyzed; therefore, a Galician origin of the c.1223_1227delACAC mutations cannot be disregarded (given the proximity of Galicia to Portugal and the known intense recent emigration history of Galicia to the Americas).

Taking all the evidences together, it seems more plausible that the two mutations c.2278C>T and c.1223_1227delACACA were founded in the Galician territory instead of being brought here by migrants.

In conclusion, the data indicate that c.2278C>T and c.1223_1227delACACA *TGM1* mutations are not mutational hotspots. These two mutations most likely arose in Galicia about 2,800–2,900 y.a., although the TMRCA dates to about only 600–1,290 y.a., indicating the existence of strong genetic drift occurring in this region at that time. The estimated ages fit well with a documented demographic scenario (supported by Bayesian Coalescent inferences) that involved drastic population size reductions in the Galician population, therefore paving the ground for strong local founder effects and endogamy in local Galician areas, followed by more recent population growth but considerable isolation from the rest of the Iberian Peninsula until very recent times.

## Materials and Methods

### Ethics Statement

All patients and family members gave written consent to participate in the study approved by the Ethical committee of the University of Santiago de Compostela. The study also conforms to the Spanish Law for Biomedical Research (Law 14/2007- 3 of July).

### Families

The patients were recruited through contacts with the Spanish patient organization for ichthyosis (http://www.ictiosis.org/home/home.htm). One of the aims of this association is to create and keep updated a national register with all the Spanish patients. Moreover, we made contact with all dermatology departments in Galicia (NW Spain) *via* mailing in order to identify those patients that were not included in the patient organization register. In addition, the families included in the present study were interviewed in order to avoid collecting closely related families. The protocol included examination by a dermatologist, a medical and dermatologic history, clinical photography and collection of blood samples for DNA analysis. The mutation status was determined using bi-directional sequencing as previously described [3].

Pedigrees were reconstructed for at least three generations (Figures S1, S2, S3, S4, S5, S6, S7, S8, S9, S10) and the demographic data of patients were obtained to determine their geographic origin (Figure 1). All apparently unrelated affected families originated from the same geographical area along the west-central coast of Galicia (the district called Rías Baixas). The families were geographically mapped along the region according to their parents' birthplaces.

The control group was obtained from a representative sample of non-affected unrelated individuals covering the whole Galician territory.

### Genotyping

We originally used HapMap CEU data to choose tagSNPs spanning 10 kb upstream and downstream of *TGM1*. We took into account non-monomorphic SNPs, previously detected by sequencing in the course of the genetic test of our patients, and two common variants, c.1559A>G and c.2160C>T that were observed in the Galician control population (minimum allele frequency (MAF) of 0.01 and 0.015, respectively) and which were forced to be selected as tagSNPs using HAPLOVIEW v4.1 [21]. Altogether, twenty-one SNPs spanning 10 kb upstream and downstream of the *TGM1* region were genotyped by a combination of SNaPshot minisequencing (primers and proves sequences, as well as PCR conditions are described in Tables S2 and S3) and bi-directional sequencing on an ABI3730xl DNA Analyzer (Applied Biosystems, Foster City, CA, USA). SNaPshot results were analyzed with GeneMapper v4 Software (Applied Biosystems, Foster City, CA, USA).

**Figure 3:**
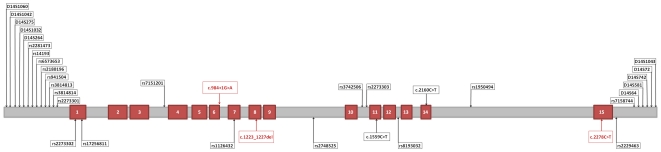
Schematic representation of the *TGM1* gene signaling the mutations, and markers analyzed in the present study. Exons are represented as red boxes.

Due to the fact that carriers shared a common SNP haplotype, the genotyped region on chromosome 14q11 was spanned to 12 Mb using ten highly polymorphic microsatellite markers (Figure 3). Microsatellite data was obtained from the UniSTS database (www.ncbi.nlm.nih.gov/sites/entrez?db=unists). Forward PCR primers were labeled with either FAM or HEX fluorescent dyes (Sigma-Genosys Ltd. Cambridgeshire, UK) (Table S4). The multiplexed amplification products were separated on the ABI3730XL and resulting data were analyzed with GeneMapper v4 Software.

All reactions were performed according to the manufacturer's protocols. Primer sequences and PCR conditions are described in Tables S1, S2 and S3.

### Haplotype reconstruction

To assign allelic phase in the families, from trio datasets, and in the 100 control individuals, the software PHASE v2.1 [22], [23] was used and double checked manually. The haplotypes for one deceased patient were inferred from the data from non-affected parents and a brother (Family 17, Figure S1 and Table S1).

### Estimation of founder mutation age

Estimation of founder mutation age was carried out using the index case from each family (see arrows in Figures S1, S2, S3, S4, S5, S6, S7, S8, S9, S10); the exceptions were the Families 2 and 17. Regarding the Family 2 (Figure S3), three independent affected chromosomes were included in the analysis because one chromosome from the maternal branch was not transmitted to the index case by his non-affected mother. Regarding Family 17 (Figure S1), the affected chromosomes were inferred from his parents and his non-affected brother given that the only affected individual from this family deceased previously to the study.

Two linkage disequilibrium based methods were used to estimate the time of the most recent common ancestor (TMRCA), Bergman's and Risch's estimators. In addition, the age of the mutations was obtained using the Bayesian method implemented in the software DMLE+2.3.

The number of generations back to the TMRCA using linkage disequilibrium based methods was first computed individually for each marker surrounding the mutation; then, the number of generations was averaged over all markers. To determinate the founder allele, and following Bergman et al., we adopted the strategy of choosing the most frequent allele outside the common region among individuals having the disease-causing mutation, hence the conserved haplotype. The physical distances were converted into centimorgans (cM) assuming a sex averaged recombination rate of 1.64 cM∼1 Mb, which was calculated by using the estimates of recombination parameters across the region in the control individuals obtained with the software PHASE. We used the Kosambi map function for translation of map distances into recombination frequencies [24].

Labuda et al. found discrepancies between the demographic and the genetic data reported in the Risch et al. study. Labuda et al. proposed a correction that is used here (instead of Risch's estimator) that accounts for the population growth rate *p*, which was estimated from the equation *N* = *N*_0_ *e* ^gp^, where *N* is the estimated present population size, *N_0_* is the estimated size of the population at the reference time and *g* is the number of generations between these two time points. The population of the Galician region at the present time is well documented by the Galician Statistical Institute (http://www.ige.eu), comprising 2,796,089 habitants. The 1591 census, which estimated the population of the six provinces comprising the Galician region, noted 629,336 habitats [12], a global figure of the Galician population in that period. Assuming 30 years per generation [25], the population growth rate was calculated to be 0.107.

The method implemented in the DMLE+ version 2.3 software package (http://www.dmle.org/), which is an extension of the Bayesian linkage disequilibrium method of Rannala and Reeve, was also used to estimate the age of the mutations, which is not necessarily the same as the age of their TMRCAs. This program was initially designed for high-resolution mapping of a disease mutation based on the observed linkage disequilibrium between the mutation and linked markers. It uses the Markov chain Monte Carlo (MCMC) method to generate the marginal posterior probability density of mutation age, based on the observed haplotypes in normal and affected chromosomes, map distances between markers and mutation site, fraction of mutated chromosomes sampled and estimated population grown rate.

The proportion of mutation-carrying chromosomes sampled was estimated on the assumptions that: (i) we genotyped all the ARCI cases in the Galician population, finding four homozygous patients for the c.2278C>T mutation and one homozygous patient for c.1223_1227del, and (ii) in the case of selection, it would operate against patients (biallelic carriers are not considered to have a heterozygous advantage or disadvantage) being relatively mild due to the low frequency of the disease. Therefore, using the Hardy-Weinberg equilibrium equation, we estimated the allele frequencies of c.2278C>T and c.1223_1227delACACA mutations in our population to be 0.1196% and 0.0598%, respectively. Therefore, the proportion of mutation-carrying chromosomes sampled was estimated to be 0.000033 and 0.000030, respectively.

A cautionary note should be added regarding the estimation of the age of founder mutations and TMRCA: (i) recombination frequencies differ in different genetic maps and human population groups, (ii) the original founder allele is unknown; and (iii) no estimates can be obtained when the founder allele frequency on control chromosomes is more common than in disease chromosomes. On the other hand, estimates based on growth rate, such as Labuda's correction and DMLE, are also a source of variability; for example, historical population data based on parish population records contain errors that are difficult to estimate. Moreover, we are assuming a constant and exponential growth rate, which could be not realistic; thus, for instance, the Galician population experienced periods of fast population growth and decreases (see above). On the other hand, the DMLE method needs data on the proportion of the population sampled, which is calculated based on the assumption of the frequency of studied mutations in the actual population and assuming Hardy–Weinberg principles. Notwithstanding, the TMRCA estimates obtained in the present study were consistent when using different statistical approaches; the age of the mutations according to DMLE showed slightly older (not overlapping) estimates than the TMRCA, which however could be consistent with the demographic history of the population (see above).

### Modeling of population history

A Bayesian Coalescent approach [26] was used to explore the historical demography of the Galician population using *TGM1* haplotipic data. This approach is implemented in the software BEAST (Bayesian Evolutionary Analysis of Sampling Trees). Unless otherwise specified default priors were used. Molecular sequences from cases and controls were used assuming that were all sampled contemporaneously. A strict clock was used and the mutation rate was set to 2.5×10^−8^ according to the average mutation rate per nucleotide site and per generation estimated by Nachman and Crowell [27]. Markov chains were run for 40,000,000 generations and sampled every 1,000 generations with the first 4,000,000 samples discarded as burn-in. The program Tracer v1.3 (http://evolve.zoo.ox.ac.uk) was used to visually inspect sampled posterior probabilities and to calculate summary statistics. The number of group sizes is an important parameter for the demographic model; this number represents the averaging of the population size estimate between the coalescent events. Given that there are no rigorous guidelines for choosing the number of groups [28], we followed the recommendation in Dodge [29]; that is, the number of groups should be set out between one and the total number of coalescent events that the tree contains minus one. Consequently, since the 217 haplotypes were grouped into 37 different clades, the number of stepwise changes in Ne was fixed to 35.

## Supporting Information

Figure S1 **Reconstructed pedigree from family 17.**
(PDF)

Figure S2 **Reconstructed pedigree from family 1.**
(PDF)

Figure S3 **Reconstructed pedigree from family 2.**
(PDF)

Figure S4 **Reconstructed pedigree from family 3.**
(PDF)

Figure S5 **Reconstructed pedigree from family 4.**
(PDF)

Figure S6 **Reconstructed pedigree from family 5.**
(PDF)

Figure S7 **Reconstructed pedigree from family 6.**
(PDF)

Figure S8 **Reconstructed pedigree from family 7.**
(PDF)

Figure S9 **Reconstructed pedigree from family 9.**
(PDF)

Figure S10 **Reconstructed pedigree from family 10.**
(PDF)

Table S1 **Patient phased haplotypes.** The most common haplotype is indicated in grey boxes. ^a^ The first Arabic number indicates the family, the Roman number the generation, the last Arabic number the affected individual, and the letter (A or B) the allele.
(PDF)

Table S2 **SNP amplification primers.** PCR was performed for a total of 40 cycles using the following conditions: 94°C denaturation for 30 s, annealing at 70°C for 3 min and extension at 72°C for 90 s, followed by 15 min of final extension at 68°C.
(PDF)

Table S3 **Minisequencing primers.** PCR was performed for a total of 25 cycles using the following conditions: 96°C denaturation for 10 s, annealing at 50°C for 5 s and extension at 60°C for 30 s. ^a^ Lower case letters denote the non-specific primer tail and letters between square brackets denote the base change.
(PDF)

Table S4 **Microsatellite primers.** PCR was performed for a total of 40 cycles using the following conditions: 94°C denaturation for 30 s, annealing at 70°C for 3 min and extension at 72°C for 90 s, followed by 15 min of final extension at 68°C. ^a^ According to GenBank Accession sequence.
(PDF)
