# Supplementary figures and images for: Multiple Local and Recent Founder Effects of TGM1 in Spanish Families
Source: PLoS One. 2012 Apr 12;7(4):e33580. doi: 10.1371/journal.pone.0033580 (PMC3325222; doi:10.1371/journal.pone.0033580)

**Figure S1.** Reconstructed pedigree from family 17.

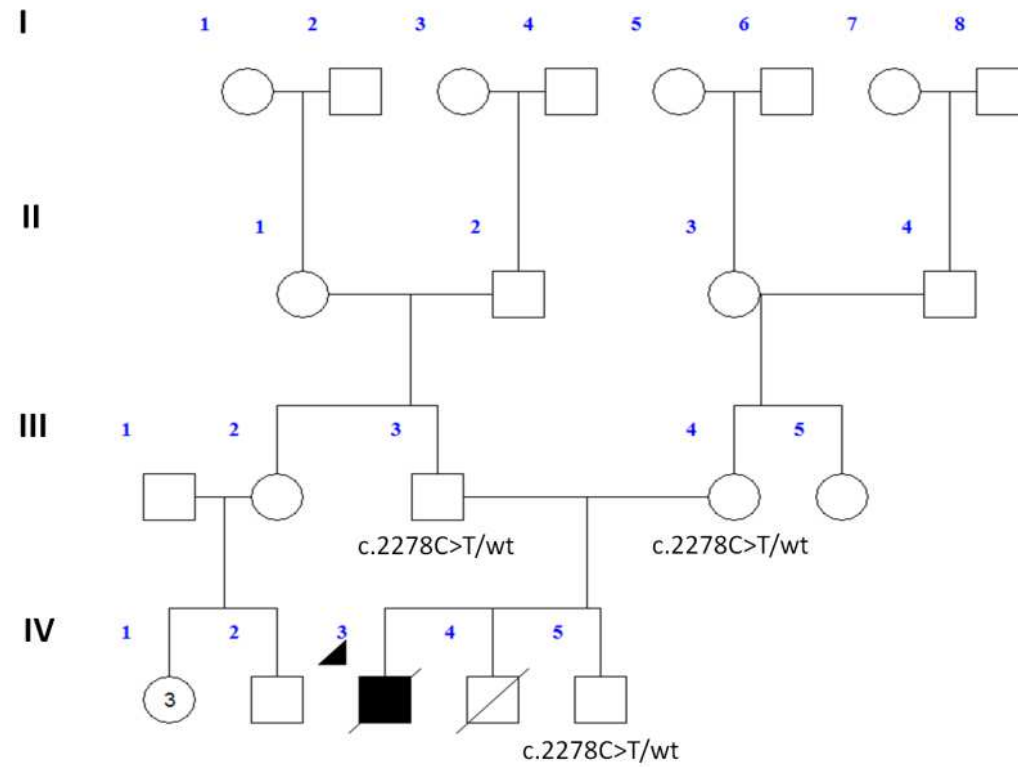

Supplement: Figure S1 — Reconstructed pedigree from family 17. (PDF) [file pone.0033580.s001.pdf]

**Figure S2.** Reconstructed pedigree from family 1.

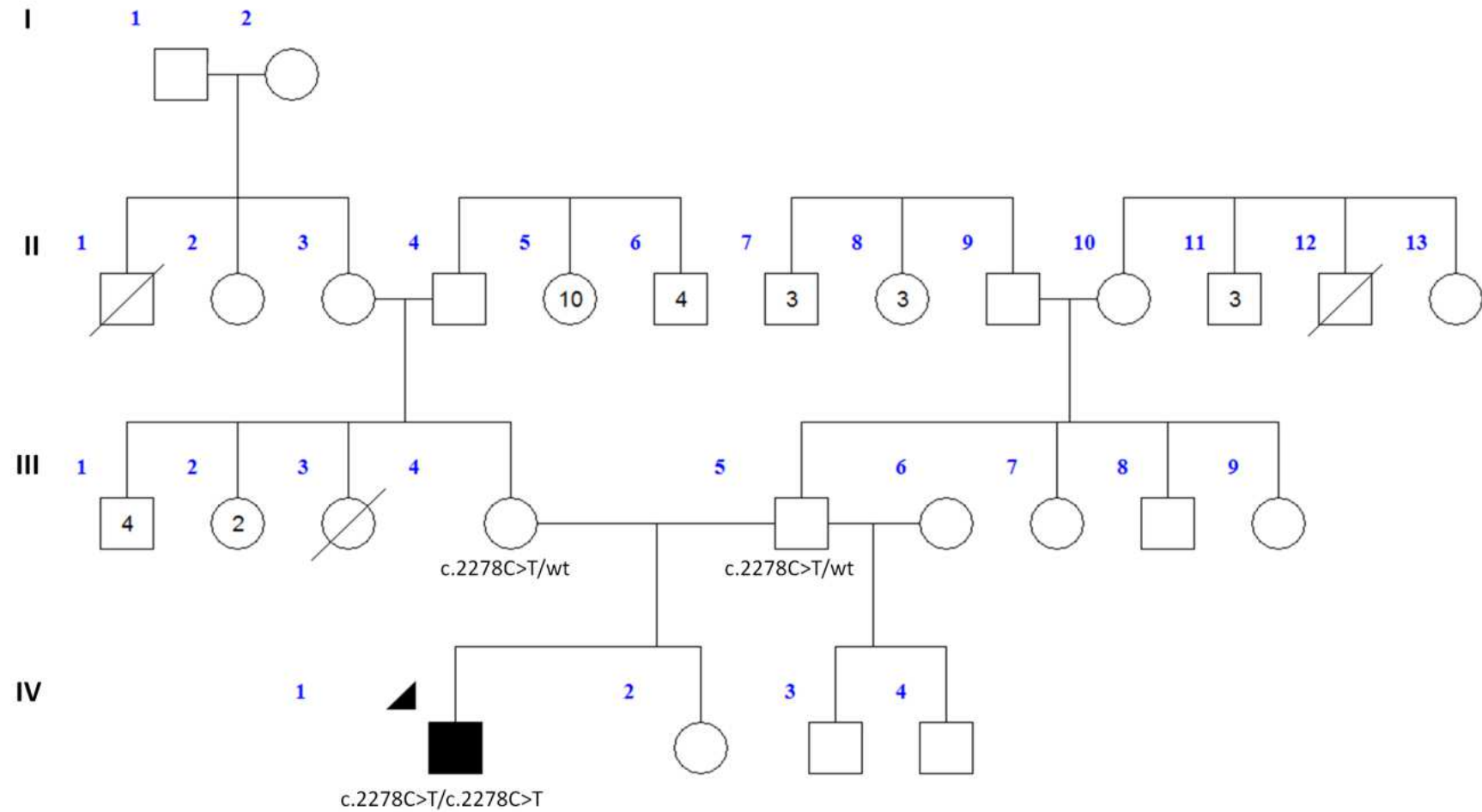

Supplement: Figure S2 — Reconstructed pedigree from family 1. (PDF) [file pone.0033580.s002.pdf]

**Figure S3.** Reconstructed pedigree from family 2.

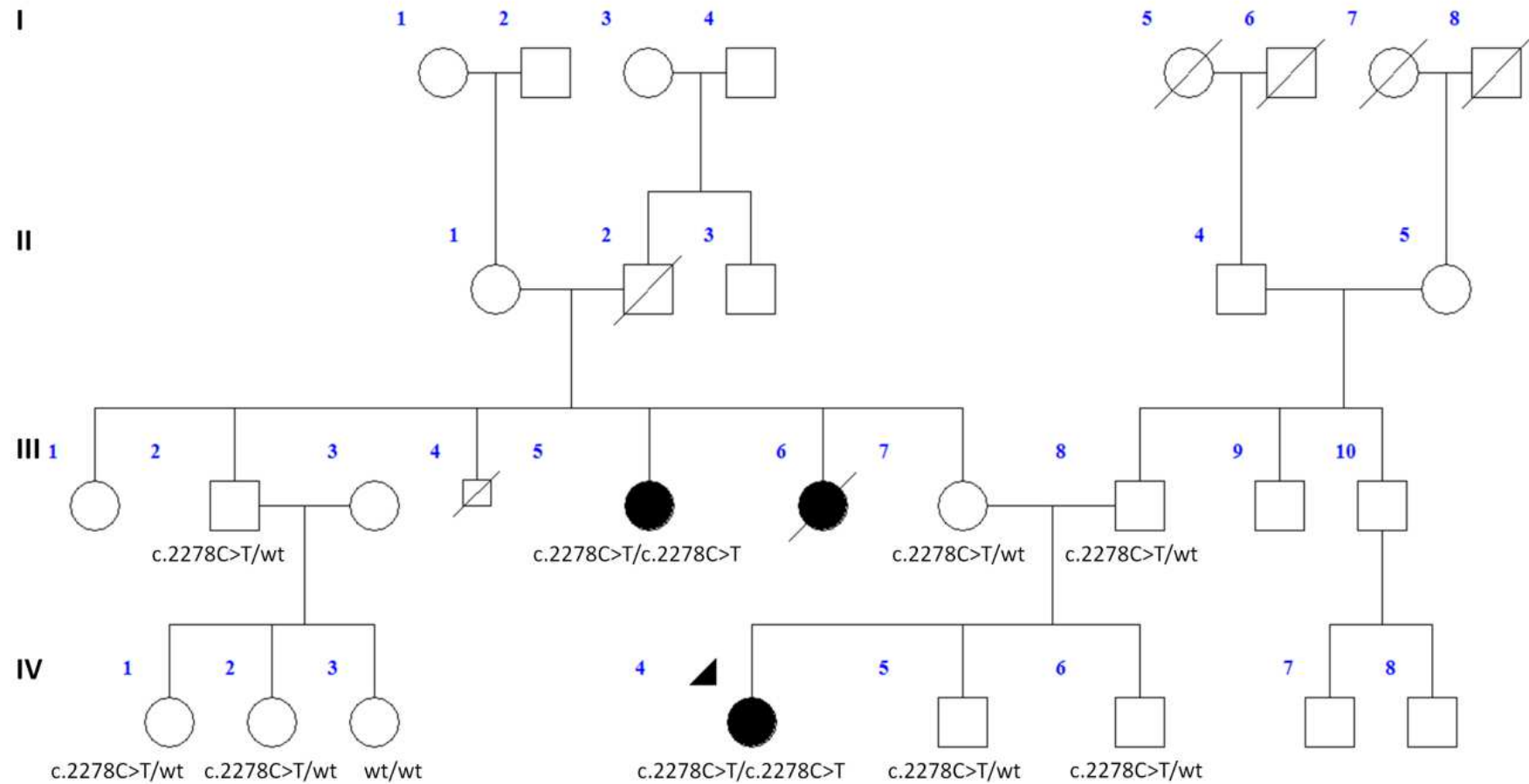

Supplement: Figure S3 — Reconstructed pedigree from family 2. (PDF) [file pone.0033580.s003.pdf]

**Figure S4.** Reconstructed pedigree from family 3.

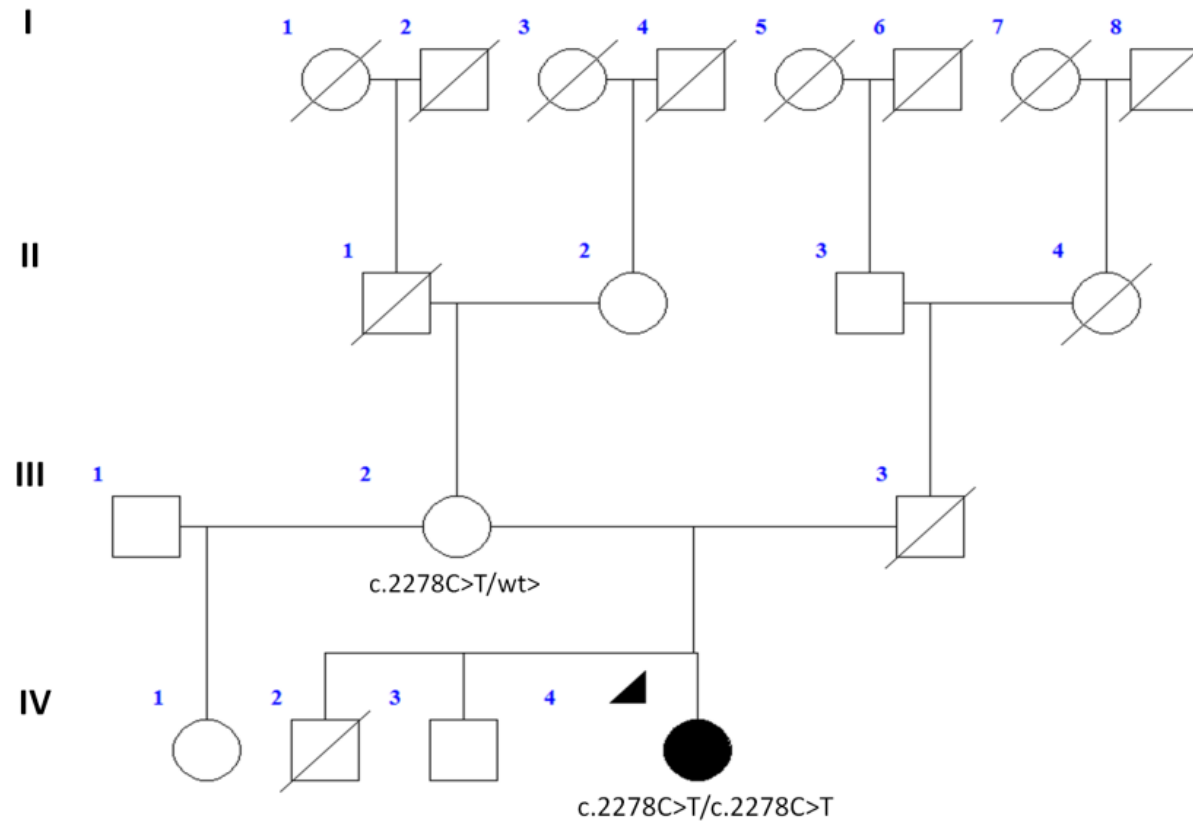

Supplement: Figure S4 — Reconstructed pedigree from family 3. (PDF) [file pone.0033580.s004.pdf]

**Figure S5.** Reconstructed pedigree from family 4.

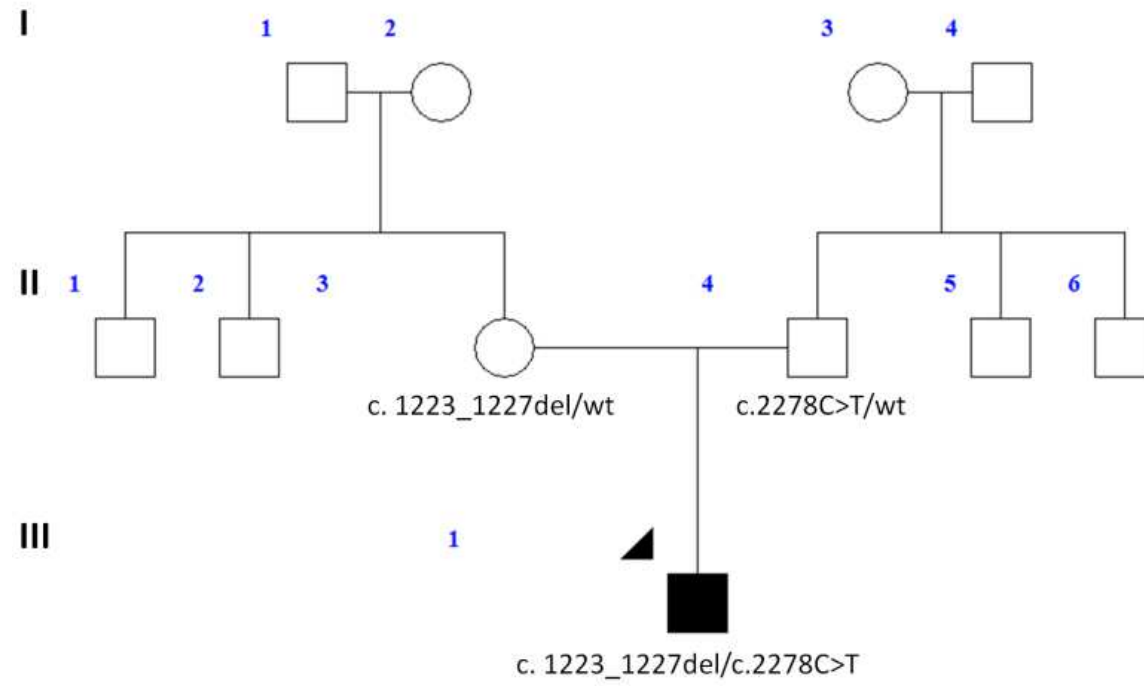

Supplement: Figure S5 — Reconstructed pedigree from family 4. (PDF) [file pone.0033580.s005.pdf]

**Figure S6.** Reconstructed pedigree from family 5.

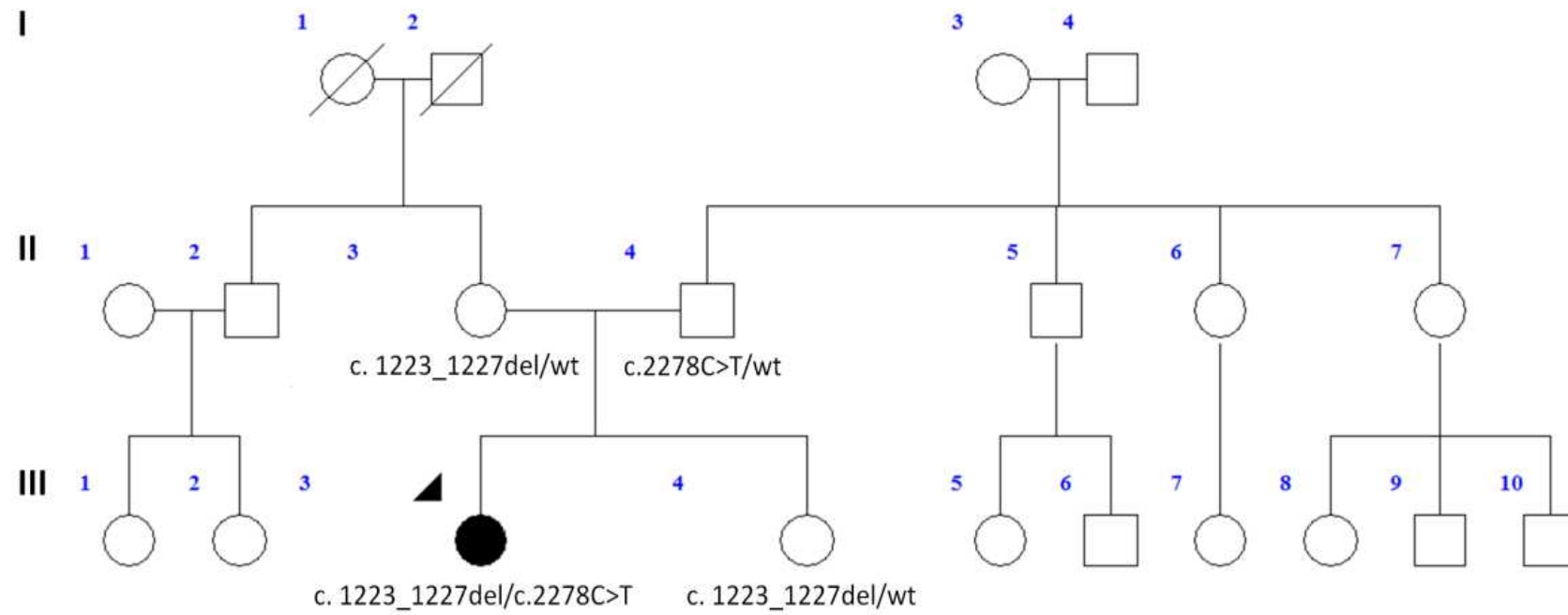

Supplement: Figure S6 — Reconstructed pedigree from family 5. (PDF) [file pone.0033580.s006.pdf]

**Figure S7.** Reconstructed pedigree from family 6.

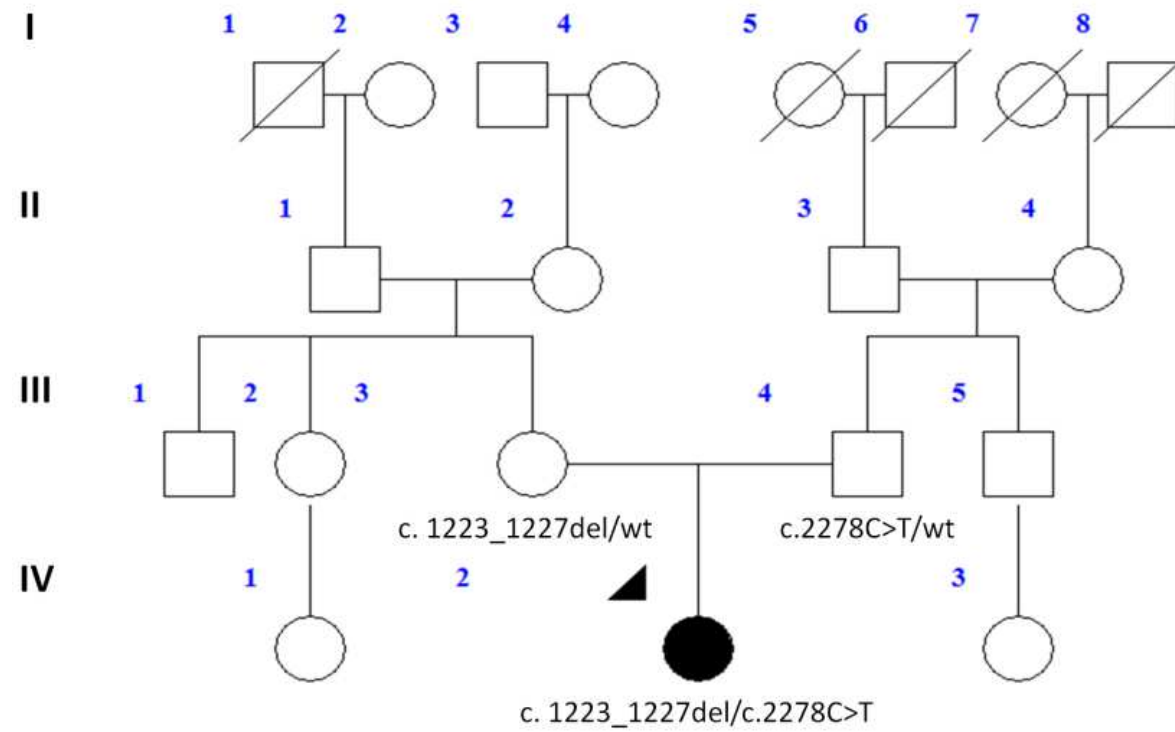

Supplement: Figure S7 — Reconstructed pedigree from family 6. (PDF) [file pone.0033580.s007.pdf]

**Figure S8.** Reconstructed pedigree from family 7.

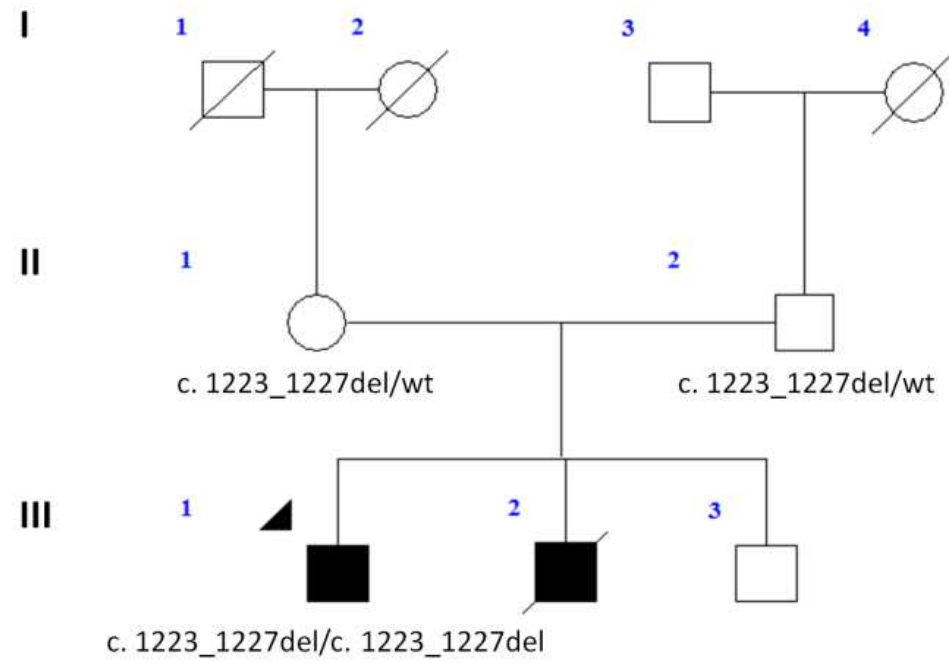

Supplement: Figure S8 — Reconstructed pedigree from family 7. (PDF) [file pone.0033580.s008.pdf]

**Figure S9.** Reconstructed pedigree from family 9.

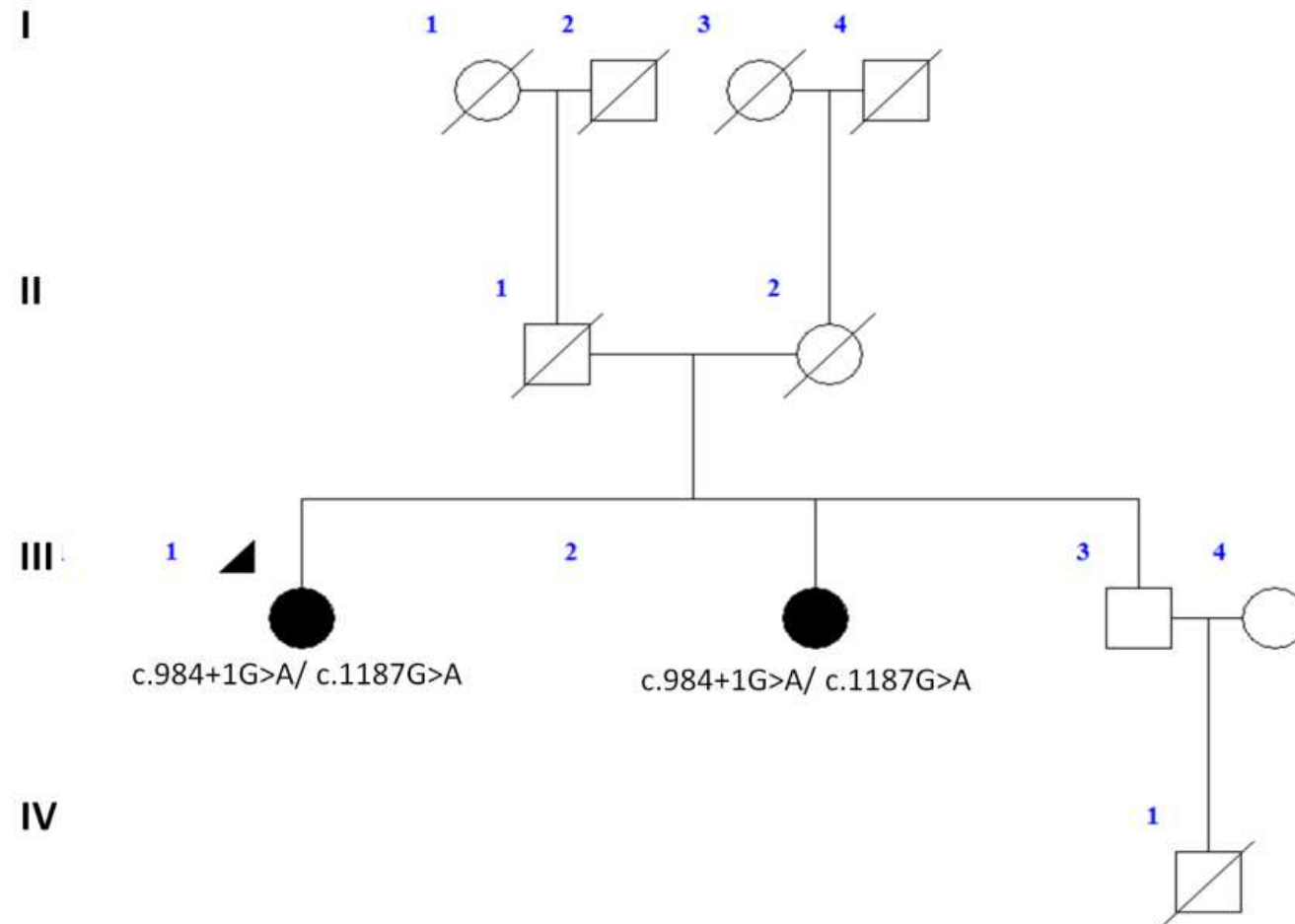

Supplement: Figure S9 — Reconstructed pedigree from family 9. (PDF) [file pone.0033580.s009.pdf]

**Figure S10.** Reconstructed pedigree from family 10.

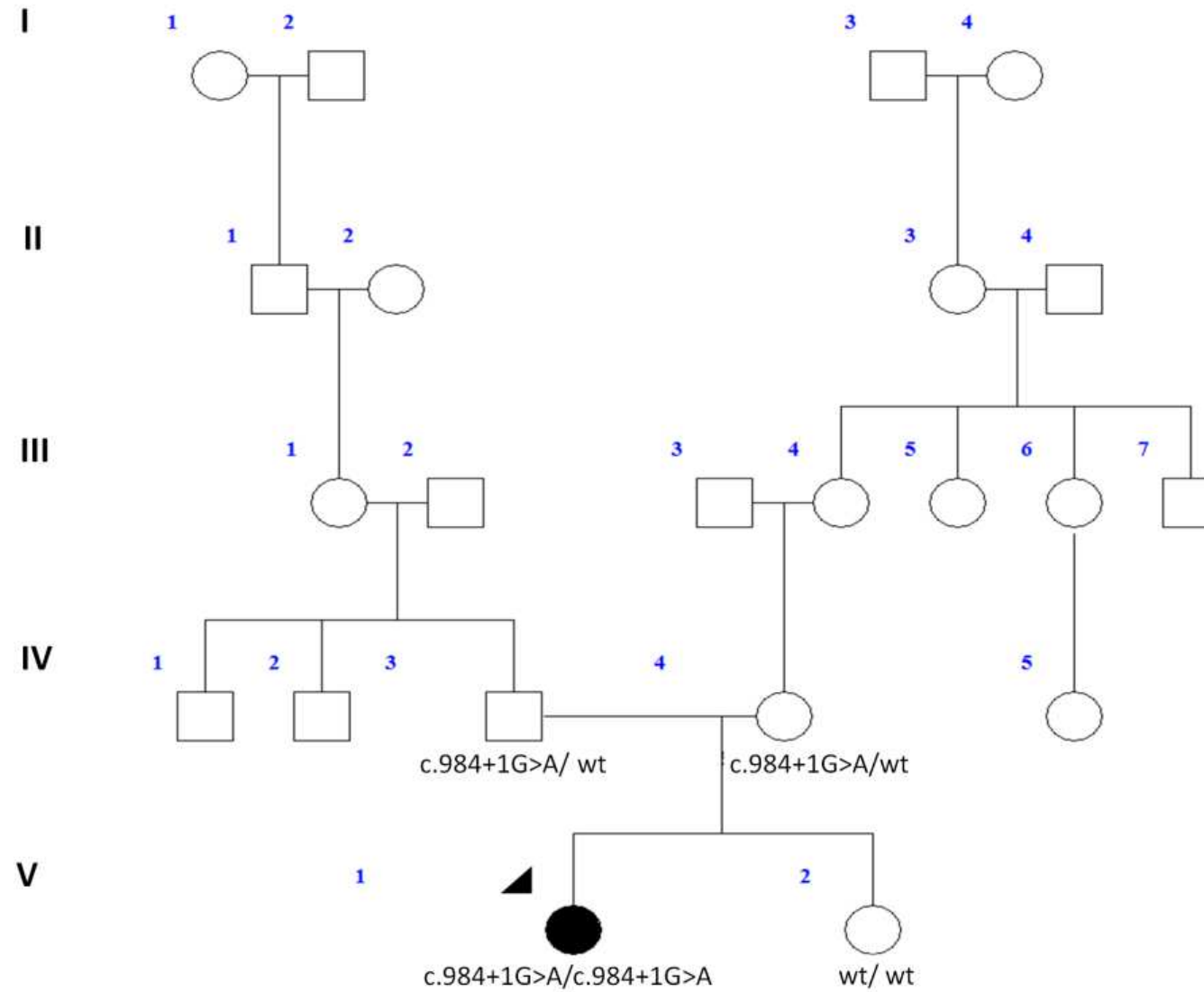

Supplement: Figure S10 — Reconstructed pedigree from family 10. (PDF) [file pone.0033580.s010.pdf]
