# Supplementary material for: Multiple Local and Recent Founder Effects of TGM1 in Spanish Families
Source: PLoS One. 2012 Apr 12;7(4):e33580. doi: 10.1371/journal.pone.0033580 (PMC3325222; doi:10.1371/journal.pone.0033580)
Supplement: Table S1 — Patient phased haplotypes. The most common haplotype is indicated in grey boxes. a The first Arabic number indicates the family, the Roman number the generation, the last Arabic number the affected individual, and the letter (A or B) the allele. (PDF) [file pone.0033580.s011.pdf]

Table S1. Patient phased haplotypes.

The most common haplotype is indicated in grey boxes. [a] The first Arabic number indicates the family, the Roman number the generation, the last Arabic number the affected individual, and the letter (A or B) the allele.

Mutation	Chr [a]	D14S1060	D14S1042	D14S275	D14S1032	D14S264	rs2281473	rs14193	rs6573653	rs2180196	rs941504	rs3814813	rs3814814	rs2273301	rs2273302	rs17256811	rs7151201	c.984+1 G>A	rs1126432	c.1223_1227 del	rs2748525	rs3742506	rs2273303	c.1559A>G	rs8193032	c.2160C>T	rs1950494	c.2278 C>T	rs2229463	rs7158744	D14S64	D14S581	D14S742	D14S72	D14S1043
c.2278C>T	1.IV.1.A	10	7	8	4	8	G	T	G	C	A	G	G	C	T	C	A	G	C	ACACA	C	G	A	A	T	C	C	T	T	T	6	2	6	3	5
c.2278C>T	1.IV.1.B	12	1	8	4	8	G	T	G	C	A	G	G	C	T	C	A	G	C	ACACA	C	G	A	A	T	C	C	T	T	T	6	2	3	7	3
c.2278C>T	2.IV.4.A	9	1	8	4	8	G	T	G	C	A	G	G	C	T	C	A	G	C	ACACA	C	G	A	A	T	C	C	T	T	T	6	2	6	2	3
c.2278C>T	2.IV.4.B	10	5	8	4	8	G	T	G	C	A	G	G	C	T	C	A	G	C	ACACA	C	G	A	A	T	C	C	T	T	T	6	2	5	7	3
c.2278C>T	2.III.5.A	10	6	7	4	6	G	T	G	C	A	G	G	C	T	C	A	G	C	ACACA	C	G	A	A	T	C	C	T	T	T	6	2	6	2	3
c.2278C>T	3.IV.4.A	10	3	5	4	8	G	T	G	C	A	G	G	C	T	C	A	G	C	ACACA	C	G	A	A	T	C	C	T	T	T	6	2	6	2	3
c.2278C>T	3.IV.4.B	4	3	5	4	8	G	T	G	C	A	G	G	C	T	C	A	G	C	ACACA	C	G	A	A	T	C	C	T	T	T	6	2	5	7	3
c.2278C>T	4.III.1.A	7	7	8	4	8	G	T	G	C	A	G	G	C	T	C	A	G	C	ACACA	C	G	A	A	T	C	C	T	T	T	6	2	5	8	3
c.2278C>T	5.III.3.A	8	1	8	4	8	G	T	G	C	A	G	G	C	T	C	A	G	C	ACACA	C	G	A	A	T	C	C	T	T	T	6	2	5	2	3
c.2278C>T	6.IV.2.A	7	6	8	4	8	G	T	G	C	A	G	G	C	T	C	A	G	C	ACACA	C	G	A	A	T	C	C	T	T	T	6	2	5	6	3
c.2278C>T	17.III.3.A	9	1	8	4	8	G	T	G	C	A	G	G	C	T	C	A	G	C	ACACA	C	G	A	A	T	C	C	T	T	T	6	2	6	3	5
c.2278C>T	17.III.4.A	7	3	7	4	6	G	T	G	C	A	G	G	C	T	C	A	G	C	ACACA	C	G	A	A	T	C	C	T	T	T	6	2	6	3	5
c.1223_1227del	4.III.1.B	10	3	6	2	5	G	C	G	C	A	G	A	C	C	C	A	G	C	del	C	G	A	A	T	T	C	C	T	T	4	1	3	5	3
c.1223_1227del	5.III.3.B	4	6	8	6	5	G	C	G	C	A	G	A	C	C	C	A	G	C	del	C	G	A	A	T	T	C	C	T	T	6	2	5	3	7
c.1223_1227del	6.IV.2.A	4	6	8	6	5	G	C	G	C	A	G	A	C	C	C	A	G	C	del	C	G	A	A	T	T	C	C	T	T	6	2	3	3	4
c.1223_1227del	7.III.1.A	10	3	6	4	5	G	C	G	C	A	G	A	C	C	C	A	G	C	del	C	G	A	A	T	T	C	C	T	T	4	2	5	2	3
c.1223_1227del	7.III.1	9	7	6	4	5	G	C	G	C	A	G	A	C	C	C	A	G	C	del	C	G	A	A	T	T	C	C	T	T	4	2	5	5	3
c.984 +1G>A	9.III.1.A	10	8	8	4	5	G	T	C	C	A	C	A	C	C	C	C	A	C	ACACA	C	G	G	G	T	C	C	C	C	C	4	2	3	7	3
c.984 +1G>A	10.V.1.A	10	3	5	4	5	G	T	C	C	A	C	A	C	C	C	C	A	C	ACACA	C	G	G	G	T	C	C	C	C	C	4	2	5	2	3
c.984 +1G>A	10.V.1.B	10	1	5	7	5	G	T	C	C	A	C	A	C	C	C	C	A	C	ACACA	C	G	G	G	T	C	C	C	C	C	4	2	3	2	3
