# Supplementary material for: Multiple Local and Recent Founder Effects of TGM1 in Spanish Families
Source: PLoS One. 2012 Apr 12;7(4):e33580. doi: 10.1371/journal.pone.0033580 (PMC3325222; doi:10.1371/journal.pone.0033580)
Supplement: Table S2 — SNP amplification primers. PCR was performed for a total of 40 cycles using the following conditions: 94°C denaturation for 30 s, annealing at 70°C for 3 min and extension at 72°C for 90 s, followed by 15 min of final extension at 68°C. (PDF) [file pone.0033580.s012.pdf]

Table S2. SNP amplification primers.

PCR was performed for a total of 40 cycles using the following conditions: 94ºC denaturation for 30 s, annealing at 70ºC for 3 min and extension at 72ºC for 90 s, followed by 15 min of final extension at 68ºC.

Multiplex	SNPs amplified	Primers	Sequence	Amplified fragment length
**Multiplex 1**	rs6573653	rs6573653F	TGACCAAGGGCTGCTGAGTGAAAAT	189
		rs6573653R	TGCGGAGTGGACTTCCTCAAGTCAG	
	rs2281473	rs2281473F	ACAGACAGTGCTGCGAGTGGGGTAA	237
		rs2281473R	TTCCTGAGAAGTTGTCCAGGCTCCA	
	rs2180196	rs2180196F	AACCTGAGCCCAAGCCCTATTCCTG	270
		rs2180196R	GCCCTCTCAGGCTACCAAGCTGTTG	
	rs7151201	rs7151201F	CCCACCTCGGGGTTGTTTCCTAGAG	330
		rs7151201R	CAGCCCAGGCACTGTGATAGTCAGG	
	rs941504	rs941504F	TGGTGCTTGGAGCTGGACAAGAAAA	353
		rs941504R	CGTCACTGGGGAAACACTGAGGATG	
	rs2748525	rs2748525F	AGGACAGGACGCCCCATCCTTTATC	381
		rs2748525R	CATTCTCCCTGCACCACCTGTCTTG	
	rs1950494	rs1950494F	CACCAGGAGAGCTGCTGTGTGTGAG	450
		rs1950494R	CTCTGCTCTTCCCACTGTGGTGCAT	
	rs7158744	rs7158744F	CCCATGAGCCCTTTGTCTTGAAAGC	539
		rs7158744R	TGCACATGGCCTTCCTGTATCATCC	
	rs14193	rs14193F	GGTGGAAGGCACGCAGTTACCTGTT	592
		rs14193R	CCACCTCCATTCCTGCCAAACTCAT	
	rs3742506	rs3742506F	GGTGAGCAGGAATGAGTGAGCCAGA	625
		rs3742506R	TGTGTGGACCTTACCCAGGGTCTGA	
	rs17256811	rs17256811F	GCTCAGCAAGTCCACACCGTTCACT	676
		rs17256811R	GAATGGTCTTCGCGGAAGGTCTCTG	
**Multiplex 2**	rs2229463	rs2229463F	TCCTGGGGAGCTGCTCTGTAGTGTG	321
		rs2229463R	CACAGGGACATTGGAGGCAATGAAA	
	rs3814813 rs3814814 rs2273301 rs2273302	rs3814813F	GTGGGGGACGTGAAATGAGATTGCT	875
		rs3814813R	AGCCTAGCTGGGGCTGACAGGTACA	
	rs1126432	rs1126432F	GCTAGTCTCTTGGGGTGTGGCATCC	991
		rs1126432R	CTCCGCCCTATCTCATCCTGTCCTG	
	rs2229464 rs2273303 rs7147300 rs8193032 rs2855009	rs2229464F	AGGAGCCTCCCAGACCAGCTTCTTC	1105
		rs2229464R	TTCACTCTCTGACCACCCCTCATGC	
