# Supplementary material for: Multiple Local and Recent Founder Effects of TGM1 in Spanish Families
Source: PLoS One. 2012 Apr 12;7(4):e33580. doi: 10.1371/journal.pone.0033580 (PMC3325222; doi:10.1371/journal.pone.0033580)
Supplement: Table S3 — Minisequencing primers. PCR was performed for a total of 25 cycles using the following conditions: 96°C denaturation for 10 s, annealing at 50°C for 5 s and extension at 60°C for 30 s. a Lower case letters denote the non-specific primer tail and letters between square brackets denote the base change. (PDF) [file pone.0033580.s013.pdf]

Table S3. Minisequencing primers.

PCR was performed for a total of 25 cycles using the following conditions: 96ºC denaturation for 10 s, annealing at 50ºC for 5 s and extension at 60ºC for 30 s. a Lower case letters denote the non-specific primer tail and letters between square brackets denote the base change.

Multiplex	Extension primer	Extension primer sequences [a]	Extension primer length
**Multiplex 1**	rs2281473	ACAAAGACTGGTCCCC [C/T]	16
	rs3742506	cagtcagtcagtCAGCGGTGAAGTTGG [A/G]	27
	rs7158744	cagtcagtcagtCAGGACCAGAATATCAGGTA [C/T]	32
	rs7151201	cagtcagtcagtcagtcagtcagtCCCACATGTTAGGGC [C/A]	39
	rs6573653	cagtcagtcagtcagtcagtcagtGTTTTTGAAAATCAGGAA [C/G]	43
	rs941504	cagtcagtcagtcagtcagtcagtcagtTTCCTTGTGTCTTCTTGTC [A/G]	47
	rs2748525	cagtcagtcagtcagtcagtcagtcagtcagtACTTACCACTCTGTCCTCTC [C/T]	52
	rs1950494	cagtcagtcagtcagtcagtcagtcagtcagtcagtCCATTGTTCTTTCTCAGGA [A/G]	55
	rs14193	cagtcagtcagtcagtcagtcagtcagtcagtcagtcagtTGACTTGTCACACAATCG [C/T]	58
	rs17256811	cagtcagtcagtcagtcagtcagtcagtcagtcagtcagtcagtcagtCCAGTTGACCCAGAAA [C/T]	64
	rs2180196	cagtcagtcagtcagtcagtcagtcagtcagtcagtcagtcagtcagtcagtCCAGCTCCTTCTTTCTG [C/T]	69
**Multiplex 2**	rs2273302	GTGCCGAGTCCAGG [C/T]	14
	rs2229463	cagtCATTGCTCCTGGCAC [A/G]	19
	rs2273303	cagtcagtcagtcagtCAGAGGGTCTGAGGG [C/T]	31
	rs2229464	cagtcagtcagtcagtcagtGTTTCACTGTGCGGC [A/G]	35
	rs7147300	cagtcagtcagtcagtcagtcagtAATCTTGAAGCTGCCAT [C/T]	41
	rs3814814	cagtcagtcagtcagtcagtcagtcagtcagtAGGGCTGTGGGTGG [A/G]	46
	rs8193032	cagtcagtcagtcagtcagtcagtcagtcagtCCTGCTTCCCTACATG [C/A]	48
	rs1126432	cagtcagtcagtcagtcagtcagtcagtcagtcagtCTACCTGTGGTGGTCAC [T/G]	53
	rs2273301	cagtcagtcagtcagtcagtcagtcagtcagtcagtcagtTCTTCTCCCCATTTCC [C/T]	56
	rs2855009	cagtcagtcagtcagtcagtcagtcagtcagtcagtcagtcagtGCCATGCTGCTCAAT [A/G]	59
	rs3814813	cagtcagtcagtcagtcagtcagtcagtcagtcagtcagtcagtcagtCCTGCTTCCCTACATG [C/G]	64
