# Supplementary material for: Multiple Local and Recent Founder Effects of TGM1 in Spanish Families
Source: PLoS One. 2012 Apr 12;7(4):e33580. doi: 10.1371/journal.pone.0033580 (PMC3325222; doi:10.1371/journal.pone.0033580)
Supplement: Table S4 — Microsatellite primers. PCR was performed for a total of 40 cycles using the following conditions: 94°C denaturation for 30 s, annealing at 70°C for 3 min and extension at 72°C for 90 s, followed by 15 min of final extension at 68°C. a According to GenBank Accession sequence. (PDF) [file pone.0033580.s014.pdf]

Table S4. Microsatellite primers.

PCR was performed for a total of 40 cycles using the following conditions: 94ºC denaturation for 30 s, annealing at 70ºC for 3 min and extension at 72ºC for 90 s, followed by 15 min of final extension at 68ºC. [a] According to GenBank Accession sequence.

Microsatellite	GenBank Accession	Primer	Secuence	Labeled by	Product size [a]
D14S64	Z16540.1	D14S64F	AGCCTGGGCAACACAGTGAGA	Fam	137
		D14S64R	CATGGGATAGAAGCAACACAGATGA		
D14S1032	Z53884.1	D14S1032F	CCTGGGTGAAGAAATAATCTGTACAACA	Hex	162
		D14S1032R	CCATGTTGCCACAAATAACAGGATT		
D14S581	G09040.1	D14S581F	TGAGGCCCCTAAGAAACCATTACAT	Fam	181
		D14S581R	CCTCCTGTGCTTACTCCTCACATTC		
D14S275	Z24107.1	D14S275F	AAGCATTCCCTATTCTGGACACCAA	Hex	193
		D14S275R	AGTATCAGGTTCTTGGGTACTCAATCTCT		
D14S1060	Z51690.1	D14S1060	TGACATGTTAAAATGGGCCACAATA	Fam	213
		D14S1060	AAAGAACTGTTATGTATCAGACCAACCCTA		
D14S264	Z23348.1	D14S264F	AGCCCCAAATATCACTCCAAATAGA	Hex	227
		D14S264R	AGAGTTGGCAACCACTTCTGTTCTTG		
D14S72	Z16878.1	D14S72F	TGGAGACTTACAGGAATAATGATCAAGC	Fam	264
		D14S72R	GGCTATCTCAGACACTTTGCAATCTATG		
D14S1042	Z50925.1	D14S1042F	CAAAGAAAGGTAAATTACTTGTGCATAACC	Hex	281
		D14S1042R	CAATTACAGGCACCTGCCATCA		
D14S1043	Z51382.1	D14S1043F	TCAGGGGCTTGGAATTATCACATAG	Fam	367
		D14S1043R	TCTGATACTCTGGAGCATAGGTATGTGTG		
D14S742	G10057.1	D14S742F	TAGGGCGTGGTTTTCTTTCATAACC	Hex	399
		D14S742R	TGCCCTCTAACATCAGTGCTTCTAA		
